# A Scoping Review on Minimum Foot Clearance: An Exploration of Level-Ground Clearance in Individuals with Abnormal Gait

**DOI:** 10.3390/ijerph181910289

**Published:** 2021-09-29

**Authors:** Abdulrahman Al Bochi, Ghazaleh Delfi, Tilak Dutta

**Affiliations:** 1 KITE—Toronto Rehabilitation Institute, University Health Network, 550 University Avenue, Toronto, ON M5G 2A2, Canada; aalbochi@ryerson.ca (A.A.B.); ghazaleh.delfi@mail.utoronto.ca (G.D.); 2 Institute of Biomedical Engineering, University of Toronto, 164 College Street, Toronto, ON M5S 3G9, Canada

**Keywords:** minimum foot clearance, minimum toe clearance, older adults, falls, tripping, prevention

## Abstract

*Background:* Falls are a major health concern, with one in three adults over the age of 65 falling each year. A key gait parameter that is indicative of tripping is minimum foot clearance (MFC), which occurs during the mid-swing phase of gait. This is the second of a two-part scoping review on MFC literature. The aim of this paper is to identify vulnerable populations and conditions that impact MFC mean or median relative to controls. This information will inform future design/maintenance standards and outdoor built environment guidelines. *Methods*: Four electronic databases were searched to identify journal articles and conference papers that report level-ground MFC characteristics. Two independent reviewers screened papers for inclusion. *Results*: Out of 1571 papers, 43 relevant papers were included in this review. Twenty-eight conditions have been studied for effects on MFC. Eleven of the 28 conditions led to a decrease in mean or median MFC including dual-task walking in older adults, fallers with multiple sclerosis, and treadmill walking. All studies were conducted indoors. *Conclusions:* The lack of standardized research methods and covariates such as gait speed made it difficult to compare MFC values between studies for the purpose of defining design and maintenance standards for the outdoor built environment. Standardized methods for defining MFC and an emphasis on outdoor trials are needed in future studies.

## 1. Introduction

Falls due to tripping are a major health concern. In fact, falling is the leading cause of injury-related hospitalizations among adults over the age of 65 years old (older adults) [1]. Every year, one in every three older adults experiences a serious fall, with trips being the most common cause of falls (33%) among the age group [2,3]. A fall can trigger a sudden downward spiral in health, leading to poor health outcomes for patients and increased healthcare costs [4].

A fall results when an individual is unable to recover from a loss of balance, notably when the individual’s center of mass moves beyond one’s base of support. This can occur as a result of a heel slip, toe slip, tripping, or turning [5].

**Figure 1:**
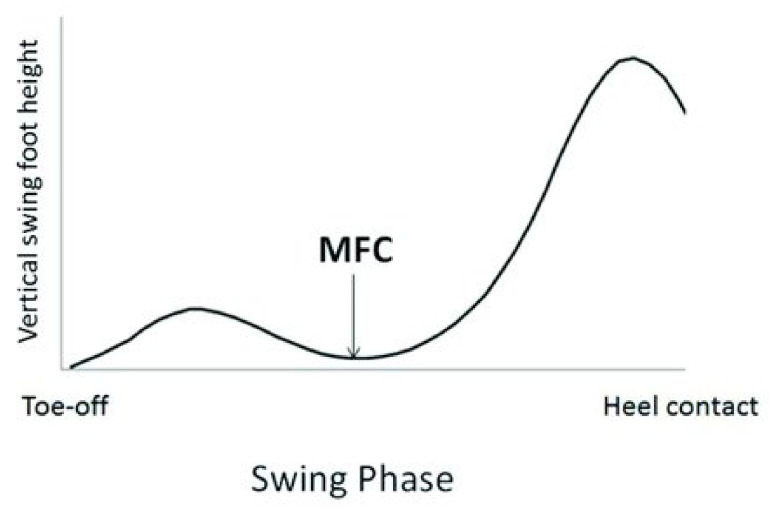
Vertical displacement of a point on the swing foot near the great toe over one stride. The minimum foot clearance (MFC) is defined by the local minima of the swing foot following toe-off. (Figure by Nagano et al. [6] licensed under CC by 4.0).

The gait cycle is comprised of two phases: the stance phase and the swing phase. The swing phase begins when the toe of the foot leaves the ground (toe-off) and continues up to the moment the heel of the same foot makes contact with the ground (heel-strike) [6]. The minimum foot clearance (MFC) point occurs approximately halfway through the swing phase and represents the local minimum distance between the swing foot and the ground (Figure 1) [7]. MFC also occurs at a time at which the horizontal velocity of the foot is maximal and the base of support is small. As a result, a low MFC value leads to a higher risk of falls due to tripping, especially on uneven surfaces or unanticipated obstacles [8].

Given that the size of a potential obstacle can have an impact on the risk of tripping, different jurisdictions impose restrictions on how large an obstacle can be. For instance, the Americans with Disabilities Act (ADA) permits vertical changes in level walkways to be a maximum of 6.4 mm. Obstacles between 6.4 and 13 mm are required to be beveled to reduce the risk of tripping [9]. In contrast, the city of Toronto guidelines allow up to 13 mm in level changes without intervention [10]. This can be particularly hazardous for vulnerable populations with low MFC values or those who have high MFC variability.

Researching MFC distributions of older adults and other vulnerable populations in real-world settings is important for understanding which populations are particularly at risk of falling due to tripping. This information can be used to inform falls prevention strategies involving the design of the built environment to reduce tripping risk. Current studies that assess MFC in individuals use various measurement systems, with the gold standard being optical motion capture systems [11]. While these systems have high accuracy, their restriction to laboratory settings limits our understanding of how gait is influenced in the real world.

To better understand the available methods to measure MFC as well as identify vulnerable populations and other conditions that impact MFC, a two-part scoping review was undertaken. The specific research questions (RQ) we set out to answer were as follows:

RQ1: What sensing modalities have been used for MFC measurement other than optical motion capture systems?

RQ2: What are the reported level ground MFC values for ambulatory adults with functional limitations, and what are the most common measurement modalities used in these assessments?

In this paper, we explore the second research question (RQ2) as we continue the two-part investigation of MFC in the scientific literature. In particular, we examine conditions that impact MFC, common research methods used in these studies, and gaps in the literature. Please refer to the companion paper for the exploration of the first research question (RQ1) [12].

## 2. Materials and Methods

This scoping review was written in accordance with the Preferred Reporting Items for Systematic Reviews and Meta-Analysis (PRISMA) scoping review reporting guidelines. A scoping review was chosen because it is the most appropriate means to accomplish the broad goal of identifying all conditions in the literature that lead to an impact on MFC mean or median.

### 2.1. Information Sources and Search Strategy

A search was conducted by one reviewer in July 2019 in the following four databases to collect potentially relevant papers: Medline, Embase, Compendex, and Web of Science collection. The search was not complemented with hand-searching or reviewing of reference lists.

The search string used was “((foot or toe? or heel?) adj2 (clear* or trajector*))” and was limited to studies written in English involving human participants. This search string was created for Medline and adapted for the other databases based on their formatting requirements. A librarian/information specialist with experience setting up knowledge synthesis projects reviewed the search strategy.

### 2.2. Eligibility Criteria

The inclusion criteria for our review were: (1) journal articles and conference papers; (2) studies that assessed MFC on level-ground surfaces. This was to ensure maximal uniformity, hence allowing for the potential to compare MFC measurements among different studies. Papers that reported the complete gait trajectory instead of only MFC values were also included given that determining MFC from these data would be possible. Given that the search was conducted in July 2019, any papers published prior to this date were included.

Reasons for exclusion included (1) children as participants, (2) stair and obstacle walking, (3) walking on sloped surfaces, and (4) studies focusing on interventions. Papers were also limited to those written in English.

Papers corresponding to RQ1 were articles that explored novel modalities or devices to measure MFC and other gait parameters. This paper reports on RQ2, which is comprised of articles that (1) reported MFC measurements of people with pathological or abnormal gait, and (2) articles with participants who were older adults or people with conditions (i.e., Parkinson’s, obesity) that may impact gait.

### 2.3. Selection of Papers

Once the initial search was completed, two reviewers (AA and GD) independently conducted abstract and full-text screening. A 3rd reviewer (TD) was consulted for resolution in case of any conflicts. The eligibility criteria proposed in Section 2.1 were followed during all levels of screening. Papers were proactively placed into two categories corresponding to each of the two research questions, RQ1 and RQ2. Title/abstract and full text screening were conducted in Covidence.

### 2.4. Data Charting and Analysis for RQ2

Both reviewers extracted the following features for articles corresponding to RQ2: condition assessed, condition effect, use of minimum toe clearance (MTC)/minimum heel clearance (MHC)/MFC, mean/median MFC/MTC/MHC values, treadmill/over ground, lab/outdoors, walking speed, gender, age, sample size, use of footwear, measurement system, marker position, and method of defining MFC/MTC/MHC. Microsoft Excel was used for the charting process. For data analysis, a forest plot was created when possible in Review Manager (Revman, v5.4) to compare studies reporting on the same condition. Cohen’s d effect sizes (with 95% confidence intervals) were calculated using the Revman software. The overall effect size for a given condition was determined according to the random-effects model given the lack of uniformity among studies. Articles that did not provide MFC values or only reported median MFC values were not included in the forest plots, because mean MFC and SD values were needed to generate Cohen’s D values. Median to mean transformations were not conducted.

The data charts were split into the following four categories to better organize the data: conditions that (i) increase mean or median MFC, (ii) decrease mean or median MFC, (iii) have no effect on mean or median MFC, and (v) “other”. Conditions that led to a statistically significant positive or negative overall effect size were placed in the “increase mean or median MFC” or “decrease mean or median MFC” categories, respectively. Conditions where the overall effect size did not achieve significance were placed in the “no effect” category. The “other” category referred to conditions where papers did not yield MFC mean or median values but instead focused on MFC variability.

### 2.5. Critical Appraisal of the Studies

A ranked critical appraisal system was not used to analyze the papers, given that the goal of the review was to simply capture all conditions that impact MFC mean or median in the literature.

## 3. Results

### 3.1. Search and Selection of Articles

**Figure 2:**
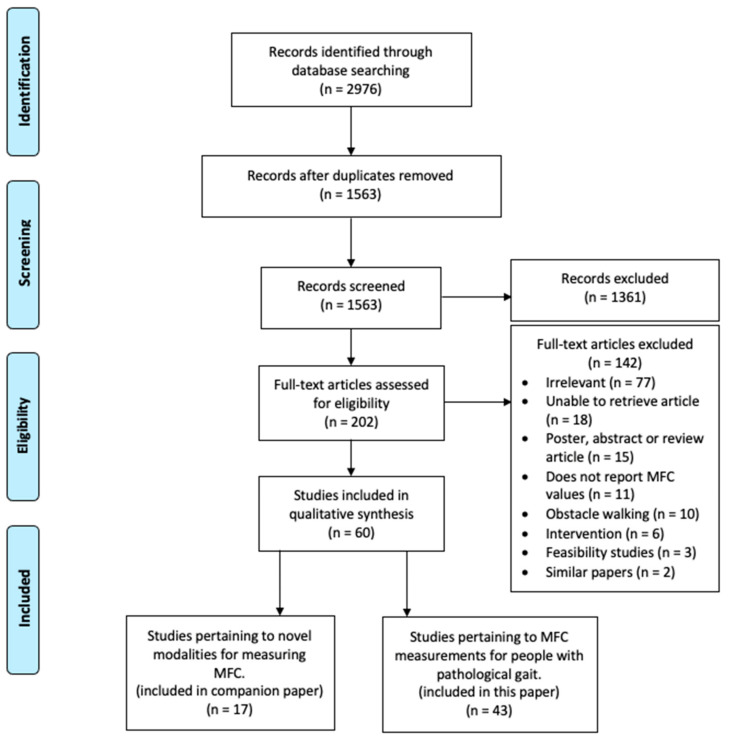
PRISMA flow diagram of study selection process.

The initial search of electronic databases conducted in July 2019 yielded 2976 journal articles and conference papers. Upon deduplication, abstract screening, and full-text screening, 43 articles matched our inclusion criteria. A flowchart outlining the selection of articles from identification to final inclusion is shown in Figure 2.

Many articles excluded at the abstract and full-text screening stages measured MFC on inclined surfaces, stairs, or through obstacles and thus did not meet our inclusion criteria of studies assessing level-ground MFC (*n* = 10). Other reasons for exclusion included papers not reporting specifically on MFC (*n* = 11) and papers focusing on interventions for preventing falls (*n* = 6). The full texts of eighteen articles could not be accessed and thus were not analyzed past abstract screening.

### 3.2. Characteristics of Included Articles

Articles ranged between 1991 and 2019 in the publication year, with the mean published in 2013. Thirty-three of the 43 included articles were written in the last 10 years. The great majority of papers were journal articles, with only two papers being conference proceedings [13,14].

#### 3.2.1. Study Design Characteristics

Of the 43 papers included in this review, an average of 41.4 participants were recruited with an average male to female ratio of 1.071 (see Table 1). Seven studies did not report the gender of participants. Over one-third of the studies conducted trials on a treadmill, while 60.5% completed trials over ground. Two studies included a combination of over ground and treadmill trials [13,15]. All studies were conducted in an indoor setting, and 79.0% of studies required participants to wear shoes as opposed to walking barefoot.

While the majority of studies used an optical motion capture system (motion capture) to measure MFC (69.8%), other measurement systems included inertial measurement units (IMUs) [16,17,18,19,20,21] and electromagnetic tracking device systems [22,23]. Three studies used a combination of motion capture with a GAITRite instrumented sidewalk [24,25,26]. One study used a combination of motion capture and IMUs [17]. One study used a novel device composed of an IMU, pressure sensors, and vibrotactile actuators [27].

The included studies also varied in the use of terminology to describe foot clearance. About three-quarters (74.4%) of studies specifically measured “MTC”, while the rest measured “MFC”.

#### 3.2.2. Areas of Research/Characteristics of Conditions Assessed

**Figure 3:**
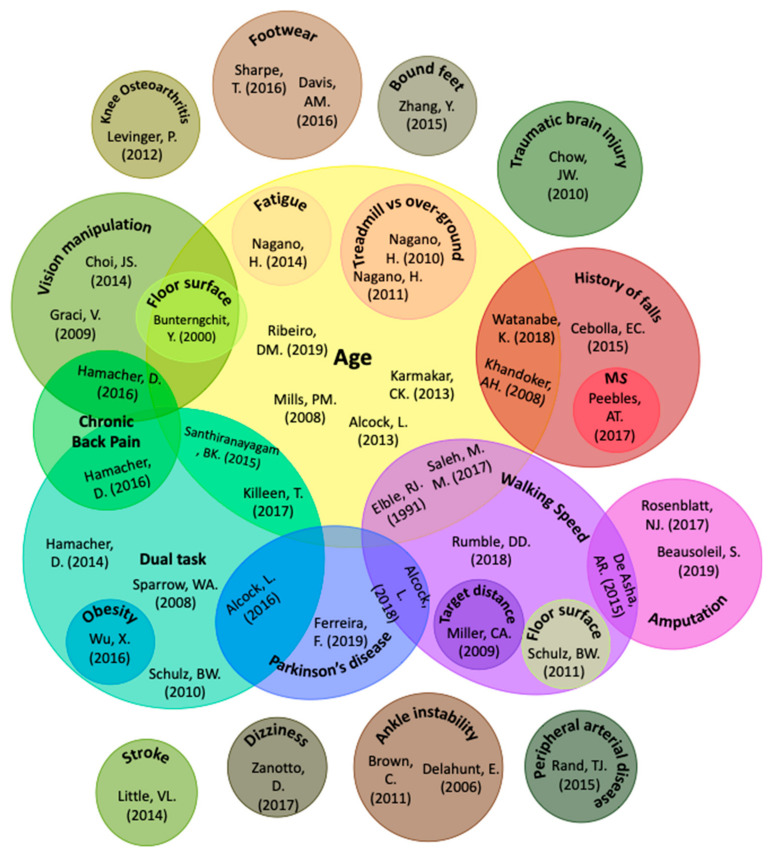
Conditions assessed in literature for effect on minimum foot clearance.

A total of twenty-eight conditions were researched for their effects on MFC among the included papers, with 53.2% of the studies involving older adults as participants. Notable conditions assessed were dual-task walking (17.0%), young vs. old gait (12.8%), gait speed (10.6%), and history of falls (6.38%). As shown in Figure 3, many of the included papers studied the combined effects of more than one condition.

##### Conditions without an Impact on MFC Mean or Median

Of the 28 conditions included in this scoping review, nine did not show a significant impact on mean or median MFC relative to controls. These included older adults relative to younger adults, Parkinson’s disease patients, older adults with a history of falls, and people with transtibial amputation. However, older adults, peripheral arterial disease patients, and older adults with a history of falls, all exhibited greater MFC variability relative to controls. Table 2 shows results for each article and condition assessed in this category. For additional detailed results, please see Table S1.

**Figure 4:**
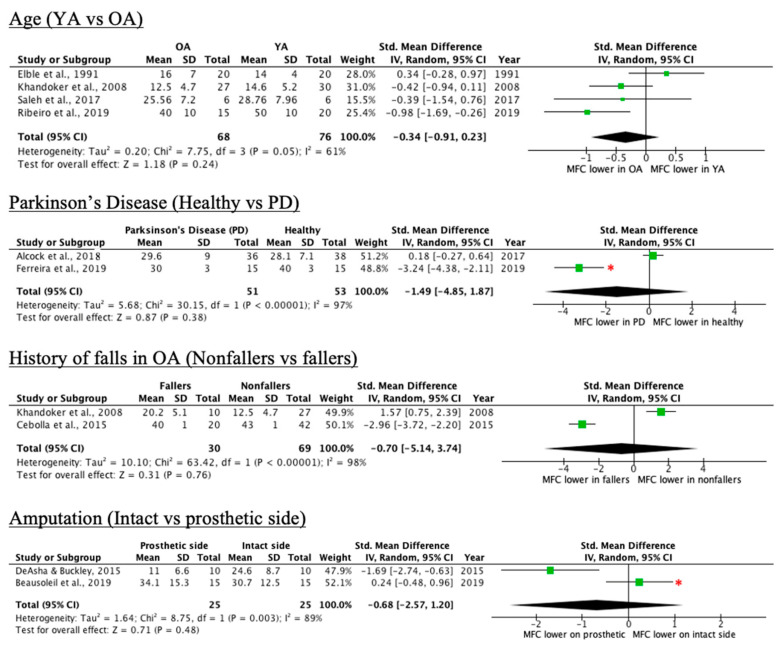
Forest plots of conditions comprising two or more studies without an overall MFC mean effect relative to control (* = original manuscript reports different finding; OA = older adults, YA = younger adults, PD = Parkinson’s disease).

**Figure 5:**
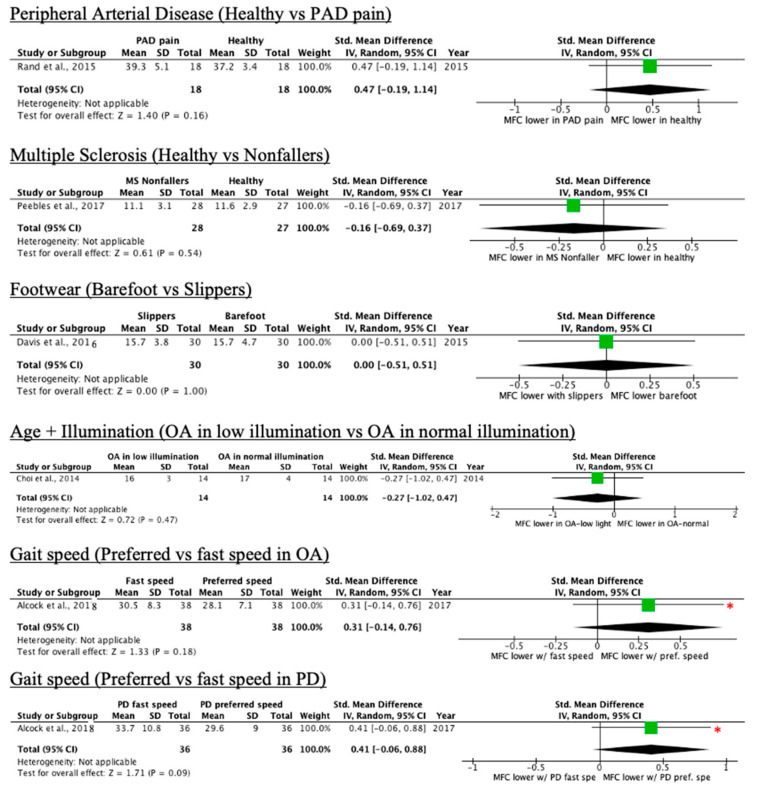
Forest plots of conditions comprising one study without an overall MFC mean effect relative to control (* = original manuscript reports different finding; OA = older adults, YA = younger adults, PD = Parkinson’s disease).

Forest plots with effect sizes and 95% confidence intervals for each condition in the “no effect” category are provided in Figure 4 and Figure 5. There are three key components of forest plots. First, each study corresponds to an effect size (shown as a green box) with whiskers corresponding to 95% confidence intervals. Second, the diamond at the bottom of the plot indicates the overall effect size when the individual studies are combined and averaged. Third, the center *y*-axis is the line of no effect. Studies where the complete box and whisker are to the right of the line of no effect are said to have a positive effect size. In the case of this paper, it signifies that the MFC mean is significantly lower in the control group as compared to the intervention group. Studies where the box and whisker are to the left of the line of no effect indicate the opposite trend. If the whisker crosses the line of no effect, then one can suppose that there is no significant difference between the control and intervention groups.

Whereas Beausoleil et al. [18] and Alcock et al. [26] reported significantly lower mean MFC in the control group, the 95% confidence intervals cross the line of no effect in the forest plots, suggesting the lack of sufficient mean difference between the control and intervention group. The opposite was observed with Ferreira et al. [19], where the authors reported no significant difference. The overall effect size for all conditions in Figure 4 and Figure 5 are non-significant.

##### Conditions That Increase MFC Mean or Median

Five of the 28 conditions led to a higher MFC relative to controls, as shown in Table 3. This included peripheral arterial disease patients without pain, people with knee osteoarthritis, participants with fitted footwear, post-stroke patients, and participants with circumferential–peripheral visual field occlusion. More detailed results can be found in Table S2.

**Figure 6:**
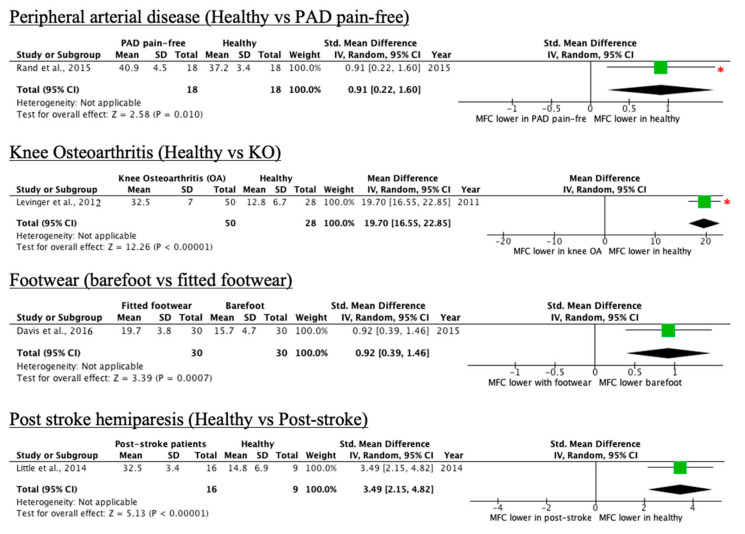
Forest plots of conditions comprising one study with an increase in MFC mean effect relative to control (* = original manuscript reports different finding).

As shown in Table 3, peripheral arterial disease pain-free patients exhibited significantly greater MTC variability as compared to the healthy group. Figure 6 showcases the forest plots for each condition in the category.

##### Conditions That Decrease MFC

Eleven of the 28 conditions led to a decrease in MFC, as seen in Table 4. Conditions included dual-task walking in older adults, walking-induced fatigue, fallers with multiple sclerosis (MS), individuals with trans tibial amputation who report trip-related stumbles, treadmill walking, and people with traumatic brain injury. More detailed results can be found in Table S3.

**Figure 7:**
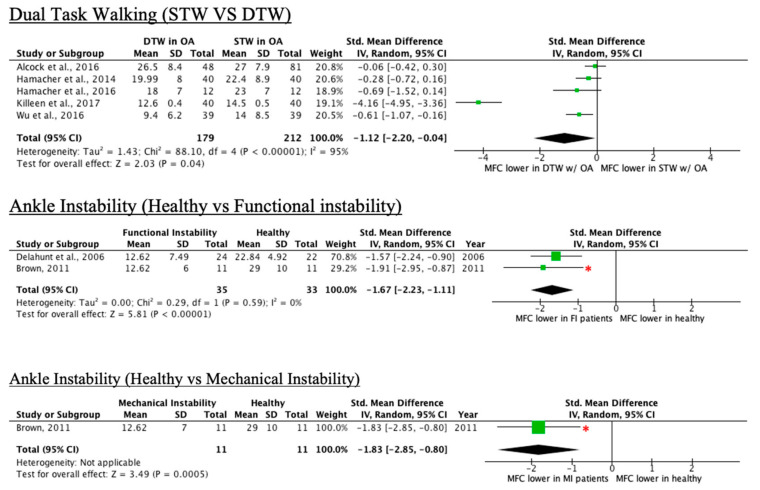
Forest plots of conditions comprising two or more studies with a decrease in MFC mean effect relative to control (* = original manuscript reports different finding; STW = single-task walking, DTW = dual-task walking).

**Figure 8:**
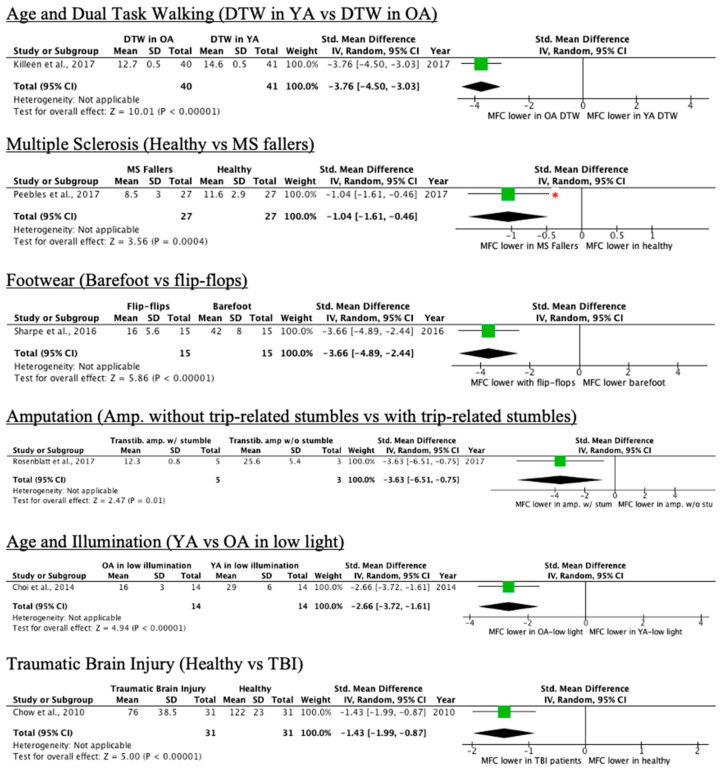
Forest plots of conditions comprising one study with a decrease in MFC mean effect relative to control (* = original manuscript reports different finding, YA = younger adults, OA = older adults).

Forest plots with effect sizes and 95% confidence intervals for each condition that decreases mean or median MFC relative to controls are provided in Figure 7 and Figure 8.

#### Other Conditions

Conditions involving papers that did not focus on MFC trends or did not fit in previous categories are listed in Table 5. For instance, for individuals with bound feet, a continuous increase in toe clearance was observed [54]. Participants with chronic back pain demonstrated significantly higher variation in MFC as compared to controls when wearing impairment goggles [20]. Detailed results can be found in Table S4.

A summary table grouping the conditions present in this paper based on their effect on MFC can be found in Table 6 below.

## 4. Discussion

The purpose of this scoping review was to explore the similarities and differences in the literature surrounding minimum foot clearance as well as to compile the reported minimum foot clearance measurements in older adults and individuals with pathological or abnormal gait. We documented the conditions assessed, MFC characteristics associated with them, and research methods used in the 43 included studies.

The results show a variety of conditions assessed in the literature, with the most comprehensive research conducted on the effect of age, history of falls in older adults, and dual-task walking on minimum foot clearance trends. Conditions that lower MFC are important to consider to better identify groups at high risk of falls. Given the presence of uneven surfaces and abrupt vertical walkway changes in the real world, studies that reflect how vulnerable populations interact with the built environment can better inform future accessibility policy and design guidelines.

### 4.1. Notable Conditions Assessed in Literature and their Limitations

#### 4.1.1. Age

When comparing gait in younger versus older adults among the included studies, it is possible to observe that aging does not significantly affect MFC mean [14,23,28,29], but that variability significantly increases in older adults [14,23,28]. This age-related increase in variability is indicative of an increased risk of falls due to tripping, given that there is a greater chance for the foot to contact the ground during swing phase [23]. Positive skewness is also present in MFC histogram distributions of older adults, which is suggestive of a motor control strategy that reduces falls risk due to a smaller variability of low MFC spread [24].

Given that an MFC histogram in older adults is positively skewed instead of normally distributed [43], it may be more suitable to investigate other MFC statistical parameters in addition to the mean.

#### 4.1.2. History of Falls

As presented in Figure 4 in the results section, while the overall effect size is not significant, there is substantial discrepancy across articles with regard to the effect of older adults with a history of falls on MFC. For instance, Cebolla et al. [32] conducted trials over ground with no footwear, whereas [28] used a treadmill and instructed subjects to wear shoes. Davis et al. [25] showed that barefoot walking leads to significantly lower MFC relative to fitted footwear. This may speak as to why the MFC values for both groups are drastically greater in [32] when compared to [28]. It is important to note that the authors in [32] did not report their method of defining MFC, which could have also impacted MFC values.

#### 4.1.3. Dual-Task Walking

While the single-task walking versus dual-task walking forest plot in Figure 7 shows an overall effect size toward MFC being lower in DTW conditions, the non-significant findings of four of the five studies [16,21,24,44] may suggest that older adults adapt to increased attention demands while maintaining habitual MFC rather than increasing it [44]. There are some limitations to note in the dual-task walking studies. Firstly, given that dual-task walking decreases gait speed [44], it is necessary to control for speed to determine the true effect of divided attention on gait. Only one study speed matched the single-task condition to the dual-task one, leading to increased variability in the single-task walking group being observed relative to the dual-task walking group [25]. Other inconsistencies among dual-task walking studies are that none of the studies employed the same dual-task. This may result in a possibility in which a given task is more attention-intensive and impacts gait patterns disproportionately among studies [47]. In addition, none of the studies reported the outcomes of the cognitive tasks. This makes walking performance biased given that there is no way to verify whether participants prioritized one task over another [16].

#### 4.1.4. Other Notable Conditions

The non-significant trend of patients with ankle instability displaying lower mean MFC when compared to controls [22,48] may suggest an increased risk for unexpected falls due to tripping. More research related to ankle instability patients is required due to the lack of studies published in this area.

For subjects with bound feet, the lack of an MTC value suggests a gait strategy in which the foot is pulled off rather than pushed off for propulsion [54]. To our knowledge, [54] is the only article looking at gait in subjects with bound feet.

In subjects with lower-limb amputation, a hip-hiking strategy may explain why higher mean MTC was observed on the amputated limb relative to the non-amputated limb [18]. Due to the lack of dorsiflexion in the affected limb, a counteracting strategy involves raising the amputated limb well above the ground [8]. This suggests that prosthesis may not replicate the gait and walking tendencies seen in healthy patients [8].

While no significant MFC difference was found between patients with Parkinson’s disease and healthy controls, researchers ought to be careful of the selection of the study cohort. For instance, Ferreira et al. [19] state that multifocal spectacles lead elderly subjects to increase MTC variability and risk of falling. Patients with late loss of vision also exhibit slower gait speed and stride length [19]. Factors such as these can negatively impact the reliability of results.

In subjects with an absence of circumferential–peripheral cues, the increase in associated MFC can be interpreted as a strategy to clear the ground more safely.

### 4.2. Research Methods Used in Studies and General Limitations among all Conditions

Our results demonstrate that a wide range of research methodologies were used in the studies we identified. This diversity serves as possible covariates and ultimately led to variation in absolute MFC values in the literature and difficulty in comparing interstudy data. Hence, the protocols and methodologies employed have the potential to become more standardized.

One inconsistency we noted among the studies we reviewed was gait speed. Fourteen out of 43 studies instructed participants to walk at a pre-selected gait speed instead of their preferred speed. Increasing gait speed beyond one’s preferred speed leads to higher MTC with a longer step length [26,38,40]. On the other hand, instructing participants to each walk at their preferred gait speed can be problematic. The difference in preferred walking speeds among subjects may influence MFC if large enough [46]. Indeed, higher gait speeds lead to increased MFC [26,38,40]. Note that some differences such as stride length and frequency can only be observed at higher gait speeds [8].

More than one-third of all of the included studies conducted experiments on a treadmill, which do not necessarily reflect the biomechanics of over-ground walking. These studies should be repeated over ground to confirm MFC characteristic trends [44]. There are many factors in treadmill walking that can impact the temporal–spatial components of gait and make direct comparison with results that employ over-ground walking difficult. For instance, in treadmill walking, the stance limb travels backward along with the treadmill belt during the instant at which MFC occurs, whereas in over-ground walking, the stance limb is fixed at one point [43]. Treadmill walking also leads to shorter stride length and stride time [55], which is correlated with a lower MFC [26]. Finally, treadmills are also shown to artificially increase gait stability while significantly reducing gait regularity during swing phase [55].

Nevertheless, treadmills are useful for measuring the value of interventions. To reduce the discrepancy in spatiotemporal parameters between over ground and treadmill walking, a treadmill familiarization period is important. Familiarization periods have also been shown to improve between-day test–retest reliability of gait measures including MFC [56]. Many of the included studies either did not report whether a treadmill familiarization period was conducted, or if mentioned, did not report the duration of the period. Meyer et al. [57] found that an acclimatization plateau for foot clearance was reached in 3.31 min. The authors also looked at 15 other walking parameters that require an acclimatization period. As a whole, all 16 walking parameters reached their acclimatization plateaus by the six-minute mark of walking, indicating this as the minimal necessary treadmill familiarization duration [57].

Upon review of the included studies, it is also evident that there is no consensus on how to measure MFC. Many authors define MFC as the minimum vertical distance between the ground and various physical markers such as the great toe [39,46,47,49], second toe [26], second metatarsal [50], fifth metatarsal [22,48,52], heel [19], and phalanx [25]. Others use virtual markers to measure MFC. For instance, [30,41,43] used the most distal point of the foot or shoe as a virtual point to measure MFC due to the difficulty in placing physical markers in that region otherwise. Some authors place markers on the unshod foot, while others place them on the footwear the participant wore. Some take the MFC measurement at 50% of the swing phase rather than seeing when the foot naturally reaches a local minimum. Seven authors did not state their method of defining MFC. The result of all of these definition discrepancies is that they contribute to differences in reported MFC values. For instance, the method chosen to define MFC in [30] led to significantly higher MFC values relative to those reported by others in the literature for the same condition. This displays the importance of having an agreed method of defining MFC in literature [43].

Terminology use associated with foot clearance such as MTC, MFC, and MHC was inconsistent. For instance, some authors use MTC to signify minimum clearance relative to the toe marker [21,46,47], while others simply use MFC [30,43,52]. MFC could also mean a variety of things as discussed above. This leads to unnecessary confusion. In addition, restricting foot clearance in reference to one fixed point on the foot eliminates the potential to find the absolute lowest point that the foot reaches relative to the ground during swing phase. A study by Telonio et al. [58] found that for obstacle clearance, the actual point of contact varies along the entire foot across subjects, so simply monitoring one point may not be indicative of true MFC.

### 4.3. Implications

The results of this study show the impact that various conditions can have on mean MFC and the associated increase in the risk of falls due to tripping. However, there are also other MFC distribution parameters and characteristics that can be explored further such as median, skewness, kurtosis, IQR, first quartile, third quartile, and maximum–minimum range [30]. This is especially important in studies with older adults due to the positively skewed MFC distribution associated with their gait [43]. While cross-sectional data are most commonly used to identify aging effects on gait, longitudinal studies can allow for confirmation of these age-related effects [30].

Given the lack of outdoor studies in this scoping review and the potential for real-world clinical gait monitoring systems in the coming years, there is an increasing need for conducting studies in an outdoor setting. This will allow one to confirm whether any gait-related changes for various conditions are impacted in an outdoor environment. However, it is important to be mindful of the challenges that outdoor studies pose such as drift errors in IMUs, the lack of a standardized walkway, and environmental factors.

While many conditions have been assessed, there is potential for further research on other aspects or conditions that affect MFC. Some conditions such as multiple sclerosis, post-stroke hemiparesis, peripheral arterial disease, knee osteoarthritis, and dizziness warrant further research due to the lack of sufficient studies.

Studies that broaden foot clearance to refer to the absolute lowest point that the foot reaches relative to the ground during the swing phase should be explored while keeping in mind that inversion or eversion of the foot may influence its precepted angle in the sagittal plane. It is also important to pay attention to possible covariates inherent in studies that may impact results with a greater emphasis on generating more standardized guidelines.

### 4.4. Recommendations for Future Research

One of the main goals of this scoping review was to learn how people with abnormal gait interact with the built environment based on their foot clearance. Designing studies that most closely simulate the real world can help better inform outdoor built environment design and maintenance standards/guidelines. As such, we have collected comments from the above sections and compiled a list of considerations that all foot clearance researchers should undertake to have data most useful for the design of the built environment. Uniformity in research methods can allow for better comparisons among different studies and the ability to synthesize new conclusions.

First, there is a need for a greater emphasis on outdoor over-ground studies to ensure findings reflect the real-world outdoor environments. Treadmill-based studies should include a familiarization period of at least six minutes as proposed by Meyer et al. [57]. Given that MFC can occur at any point on the foot throughout the swing phase [58], we propose that MFC should be measured relative to multiple points on the foot. This allows for obtaining the absolute lowest MFC point, but being mindful if measurements are only taken relative to one axis. Allowing participants to walk at their own pace and to wear shoes also allows for data that reflects how people would interact with the built environment in a real-world setting. Some of these recommendations will likely increase the variability in MFC values and be seen as problematic because it may make it more challenging to find statistically significant differences between groups. However, it is important to note that capturing more realistic MFC variability is critical when using these data to define standards for the built environment.

### 4.5. Limitations of this Scoping Review

Journals were not hand-searched through checking reference lists, and unpublished or gray literature were not searched. Although articles included in the scoping review were analyzed on their research methods, they were not subjected to a ranked critical appraisal system.

In addition, some conditions may not have been appropriately categorized in terms of their effect on mean or median MFC due to the lack of sufficient studies on that topic.

## 5. Conclusions

While the quality of the included studies was found to be relatively high, the lack of standardized research methods made it difficult to compare MFC values between studies. Covariates such as gait speed, treadmill versus over ground, and familiarization periods all have the potential to impact MFC. More uniform methods of defining MFC and an emphasis on outdoor studies should be explored in future work to allow for findings to be used to create outdoor built environment design and maintenance standards/guidelines.

## Figures and Tables

**Table 1:** Study design characteristics in included papers.

Parameter	Value
Mean total # of participants	41.4
Mean male to female ratio	1.071
Use of treadmill	34.9%
Indoors setting	100.0%
Participants wearing shoes	79.0%
Equipment used	30 motion capture, 6 IMU, 2 electromagnetic system, 5 combination of systems
MTC to MFC	74.4% to 25.6%

**Table 2:** Summary of findings in included papers for conditions without an impact on MFC mean or median relative to controls.

Condition/IV Assessed	Control vs. Intervention	Article	MFC Mean/ Median	Impact of Condition on MFC/MTC Mean/Median	Impact of Condition on MFC Variability	MFC: Control Group (mm)	MFC: Intervention Group (mm)
Age	YA vs. OA	[14]	Mean	No sig. mean MFC diff	Sig greater in OA	28.76 ± 7.96	25.56 ± 7.20
		[28]	Mean	No sig. mean MFC diff	Sig greater in OA	14.6 ± 5.2	12.5 ± 4.7
			Median	No sig. median MFC diff	Sig greater in OA	14.4 ± 5.2	12.1 ± 4.7
		[29]	Mean	No sig. mean MFC diff	Not reported	14 ± 4	16 ± 7
		[23]	Median	No sig. median MFC diff	Sig greater in OA	14.9 ± 1.6	13.8 ± 2.1
		[30]	Mean	Sig. lower mean MFC in OA	N/A	50 ± 10	40 ± 10
		[31]	Mean	Decreases in MFC of 0.2 cm/year of age	N.R.	N.R.	N.R.
Parkinson’s disease	Healthy vs. idiopathic PD	[19]	Mean	No sig. mean MFC diff	No sig. diff	40 ± 3	30 ± 3
	Healthy vs. vascular PD			No sig. mean MFC diff	No sig. diff	40 ± 3	30 ± 2
	Healthy vs. PD (at preferred speed)	[26]	Mean	N.R.	N/A	28.1 ± 7.1	29.6 ± 9
	Healthy vs. PD (at fast speed)			N.R.		30.5 ± 8.3	33.7 ± 10.8
History of Falls in OA	Non-fallers vs. fallers	[32]	Mean	Sig. lower mean MFC in Fallers	N.R.	43 ± 1	40 ± 1
		[28]	Mean	Sig. lower mean MFC in Non-fallers	Sig. greater in fallers	12.5 ± 4.7	20.2 ± 5.1
		[33]	Median	Sig. lower median MFC in Non-fallers	Sig. greater in fallers	12.1 ± 4.7	20 ± 5.1
	Falls and time: MTC at 5–10 min vs. MTC at 15–20 min in OA	[34]	Mean	Sig. lower MTC with time in OA w/o history of falls, but not in those with one or in YA	Sig. greater MTC variability in OA than YA	Graph	Graph
Amputation	Non-amputated limb vs. amputated limb	[18]	Mean	Sig. higher mean MTC on amputated limb	N.R.	30.7 ± 12.5	34.1 ± 15.3
	Intact vs. prosthetic side	[35]	Mean	Sig. lower MTC on prosthetic side (no increase in MTC with increasing gait speed on prosthetic side)	N.R.	24.6 ± 8.7	11.0 ± 6.6
Peripheral Arterial Disease with pain	Healthy vs. PAD pain	[36]	Mean	No sig. mean MFC diff	Sig higher MTC variability diff in PAD pain patients	37.2 ± 3.4	39.3 ± 5.1
Non-fallers with Multiple Sclerosis (MS)	Healthy Control vs. MS Non-fallers	[17]	Mean	N.R.	N.R.	11.6 ± 2.9	11.1 ± 3.1
Slippers	Barefoot vs. slippers	[25]	Mean	No sig. mean MFC diff	N.R.	15.7 ± 4.7	15.7 ± 3.8
Age + Illumination	OA in normal conditions vs. OA in low illumination conditions	[37]	Mean	No sig. MTC diff b/w illumination conditions in OA (sig diff for younger group)	N.R.	17 ± 4	16 ± 3
Gait Speed	Gait Speed in OA	[26]	Mean	Higher mean MTC with increasing speed and longer step length	N/A	28.1 ± 7.1	30.5 ± 8.3
		[38]	Mean	Sig. higher mean MTC with increasing speed	No sig. diff in variability with increasing speed	N/A	N/A
	Gait Speed in Healthy YA	[39]	Mean	Sig lower mean MTC with increasing treadmill speed (Graph)	N.R.	N/A	N/A
		[40]	Mean	Sig. higher mean MTC with increasing gait speed; Sig higher mean MTC from no obstacle to visible obstacle, and from visible to hidden obstacle	No sig changes in variability across obstacle conditions	N/A	N/A
	PD preferred speed vs PD fast speed	[26]	Mean	Sig. higher mean MTC at faster speed	N/A	29.6 ± 9	33.7 ± 10.8

OA = older adults, YA = younger adults, PD = Parkinson’s disease, PAD = peripheral arterial disease, N/A = not applicable, and N.R. = not reported in the article.

**Table 3:** Summary of findings in included papers for conditions that increase mean or median MFC relative to controls.

Condition/IV Assessed	Control vs. Intervention	Article	MFC Mean/ Median	Impact of Condition on MFC/MTC Mean/Median	Impact of Condition on MFC Variability	MFC: Control Group (mm)	MFC: Intervention Group (mm)
Peripheral Arterial Disease without pain	Healthy vs. PAD pain-free	[36]	Mean	No sig. diff	Sig higher MTC variability diff in PAD pain-free patients	37.2 ± 3.4	40.9 ± 4.5
Knee Osteoarthritis	Healthy vs. KO	[41]	Mean	No sig. diff	N.R.	12.8 ± 6.7	32.5 ± 7
Fitted footwear	Barefoot vs. fitted footwear	[25]	Mean	Sig. lower MFC in barefoot walking	N.R.	15.7 ± 4.7	19.7 ± 3.8
Post-stroke hemiparesis	Healthy vs. post-stoke patients	[42]	Mean	Sig. higher MTC in Post-Stroke patients	N.R.	14.8 ± 6.9	32.5 ± 3.4
Vision obstruction	FV (full vision) vs. CPO (circumferential-peripheral occlusion), UO (upper occlusion), LO (lower occlusion)	[43]	Median	Sig. higher MFC with CPO compared to rest	N.R.	N/A	N/A

OA= older adults, YA = younger adults, PD = Parkinson’s disease, PAD = peripheral arterial disease, N/A = not applicable, and N.R. = not reported in the article.

**Table 4:** Summary of findings in included papers for conditions that decrease mean or median MFC relative to controls.

Condition/IV Assessed	Control vs. Intervention	Article	MFC Mean/ Median	Impact of Condition on MFC/MTC Mean/Median	Impact of Condition on MFC Variability	MFC: Control Group (mm)	MFC: Intervention Group (mm)
Dual Task Walking (DTW)	Single Task Walking (STW) vs. DTW in OA	[44]	Mean	No sig. mean MFC diff (GRAPH)	Sig. greater variability in preferred speed single task walking group	N/A	N/A
		[21]	Mean	No sig. mean MFC diff	No sig variability diff	22.4 ± 8.9	19.99 ± 8
		[16]	Mean	No sig. mean MFC diff	No sig variability diff	23 ± 7	18 ± 7
		[24]	Mean	No sig. mean MFC diff	No sig variability diff	27 ± 7.9	26.5 ± 8.4
		[45]	Mean and Median	No Dual Task effects; Right foot MFC sig greater than left foot for YA and OA	Sig. higher MFC variability in OA for right-foot only	Graph	Graph
		[8]	Mean	MTC changes dependent on task (↑ for carrying basket, ↓ for answering questions, and unchanged for carrying water on tray)	N.R.	Graph	Graph
		[46]	Mean	Incongruent Stroop Task: Sig lower mean MFC in DTW	No sig. variability diff	14.5 ± 0.5	12.7 ± 0.5
				Congruent Stroop Task: Sig. lower mean MFC in DTW	No sig. variability diff	14.5 ± 0.5	12.6 ± 0.4
	STW vs DTW in obese and comparison groups (combined data)	[47]	Mean	Sig. lower mean MTC in DT	N.R.	14 ± 8.5	9.4 ± 6.2
Age + Dual Task Walking (DTW)	DTW in YA vs DTW in OA	[44]	Mean	No sig. mean MFC diff (GRAPH)	No sig variability diff	N/A	N/A
		[46]	Mean	Incongruent Stroop Task: Sig. lower mean MFC in OA	Sig. greater variability in OA	14.6 ± 0.5	12.7 ± 0.5
				Congruent Stroop Task: No sig. diff (values not given)	Sig. greater variability in OA	N/A	N/A
Ankle instability	Functional instability (FI) (Control vs. FI Patients)	[48]	Mean	Sig. lower mean MFC in FI individuals	N.R.	22.84 ± 4.92	12.62 ± 7.49
		[22]	Mean	No sig. mean MFC diff	N.R.	29 ± 10	12.62 ± 6
	Mechanical instability (MI) (Control vs. MI Patients)			No sig. mean MFC diff	Sig. increased variability in MI group	29 ± 10	12.62 ± 7
Fallers with Multiple Sclerosis (MS)	Healthy Control vs. MS Fallers	[17]	Mean	Sig. lower MTC in MS Fallers	No sig variability diff	11.6 ± 2.9	8.5 ± 3
Flip-flops	Barefoot vs flip-flops	[49]	Mean	Sig. lower mean MFC in flip-flops	N.R.	42 ± 8	16 ± 5.6
Transtibial Amputation + Trip-related stumbles	No history of trip-related stumbles (TS) vs. trip-related stumbles (TS)	[50]	Mean	Sig. lower MTC (prosthetic side) for individuals that report TS	N.R.	25.6 ± 5.4	12.3 ± 0.8
Age + Illumination	YA vs. OA in low illumination conditions	[37]	Mean	Sig. lower MTC in OA	N.R.	29 ± 6	16 ± 3
Traumatic Brain injury (TBI)	Healthy (slow speed) vs. TBI (preferred speed)	[51]	Mean	Sig. lower mean MTC in TBI patients (regardless of walking speed)	N.R.	122 ± 23	76 ± 38.5
Fatigue	Non-fatigued vs. fatigued OA	[52]	Mean	Sig. lower mean MFC in fatigued OA	Sig. reduced in fatigued OA (graph)	N/A	N/A
Age + Treadmill/Overground	Overground vs. Treadmill	[15]	Median	Sig. lower median MFC in treadmill walking; non-dominant foot MFC greater than dominant foot on treadmill; no ageing effects	Lower variability on treadmill (nonsig.)	Graph	Graph
		[13]	N.R.	Sig. lower MTC in treadmill walking; greater assymetry in OA; no ageing effect	N.R.	17.5	12.1
Age + Floor surface + Vision Obstruction	YA vs. OA with and without load (i.e vision obstruction)	[53]	Mean	Age × Load: Sig. lower mean MFC in OA carrying load (i.e. w/ vision obstruction)	N.R.	Graph	Graph
	OA w/o load vs w/load while transitioning from carpet to vinyl			Age × Load × Floor: Sig lower mean MFC in OA when carrying load and transitioning (i.e. w/vision obstruction)	N.R.	Graph	Graph

OA = older adults, YA = younger adults, N/A = not applicable, and N.R. = not reported in the article.

**Table 5:** Summary of findings in included papers for conditions in “other” category.

Condition/IV Assessed	Control vs. Intervention	Article	MFC Mean/ Median	Impact of Condition on MFC/MTC Mean/Median	Impact of Condition on MFC Variability	MFC: Control Group (mm)	MFC: Intervention Group (mm)
Chronic back pain	Healthy vs chronic lower back pain (no vision obstruction)	[20]	N/A	MTC data not provided (Assessed variability)	No sig difference in coefficient of variation	N/A	N/A
	Healthy vs chronic lower back pain (LBP) (impairment goggles)				Sig higher CV of MFC for chronic LBP patients	N/A	N/A
	Chronic LBP patients with vs without visual obstruction (i.e. impairment goggles)				Sig higher CV of MFC for chronic LBP patients with visual obstruction	N/A	N/A
Bound feet	Control vs patients with bound feet	[54]	Mean	Continuous increase in MTC; MHC increased at 20% of swing phase then decreased	N.R.	N/A	N/A
Dizziness	Mean Foot clearance of cohort with a range of DHI-S scores	[27]	Mean	MTC data not provided	N.R.	N/A	32.5

Where N/A = not applicable, and N.R. = not reported in the article.

**Table 6:** All conditions compiled based on effect on MFC.

Effect on MFC	Conditions
Decrease	Dual-task walking, age and dual-task walking, ankle instability, fallers with multiple sclerosis, flip-flops, transtibial amputation + trip-related stumbles (variability ↓), age and illumination, traumatic brain injury, fatigue (variability ↓ in fatigued older adults), age and treadmill/over ground, age and floor surface and vision obstruction.
No effect	Age (variability ↑), Parkinson’s disease, history of falls in older adults, amputation, peripheral arterial disease with pain (variability ↑), non-fallers with multiple sclerosis, slippers, age and illumination, gait speed
Increase	Peripheral arterial disease without pain, knee osteoarthritis, fitted footwear, post-stroke hemiparesis, vision obstruction

## Data Availability

The data supporting the conclusions of this study are available in the manuscript and supplementary materials.

## Supplementary Materials

The following are available online at https://www.mdpi.com/article/10.3390/ijerph181910289/s1, Table S1: Extracted data from papers with conditions without an impact on MFC mean or median relative to controls, Table S2: Extracted data from papers with conditions that increase MFC mean or median relative to controls, Table S3: Extracted data from papers with conditions that decrease MFC mean or median relative to controls, Table S4: Extracted data from papers with conditions in “other” category.

## Abbreviations

CPO = Circumferential–Peripheral Occlusion; DTW = Dual Task Walking; FI = Functional Instability; FV = Full Vision; KO = Knee Osteoarthritis; LBP = Lower Back Pain; LO = Lower Occlusion; MFC = Minimum Foot Clearance; MHC = Minimum Heel Clearance; MI = Mechanical Instability; MS = Multiple Sclerosis; MTC = Minimum Toe Clearance; N/A = Not Applicable, N.R. = Not Reported; OA = Older Adults; PAD = Peripheral Arterial Disease; STW = Single Task Walking; PD = Parkinson’s Disease; TBI = Traumatic Brain Injury; TS = Trip-Related Stumbles; UO = Upper Occlusion; YA = Young Adults.
